# Stoichiometric Mismatch between Consumers and Resources Mediates the Growth of Rocky Intertidal Suspension Feeders

**DOI:** 10.3389/fmicb.2017.01297

**Published:** 2017-07-12

**Authors:** Matthew E. S. Bracken

**Affiliations:** Department of Ecology and Evolutionary Biology, University of California, Irvine Irvine, CA, United States

**Keywords:** Chl *a*, C:N, ecological stoichiometry, intertidal, marine, mussel, phytoplankton, stoichiometric mismatch

## Abstract

The concept of ecological stoichiometry—the balancing of elemental ratios in ecological interactions—has transformed our thinking about processes in natural systems. Here, this perspective is applied to rocky shore ecosystems to explore the consequences of variation in internal nutrient ratios across two trophic levels. Specifically, I measured the internal concentrations of carbon (C) and nitrogen (N) in mussels (*Mytilus* spp.) and particulate organic matter (POM) to evaluate the effects of stoichiometric mismatch—the difference in the carbon-to-nitrogen ratio (C:N) between a consumer and its resources—on mussel growth at sites on the coasts of Oregon, USA, and the South Island of New Zealand. As POM quality (i.e., Chl *a*, a proxy for phytoplankton availability in the POM) increased, C:N of the POM declined, but C:N of mussels increased. This resulted in a greater mismatch in C:N between mussels and their food source at low Chl *a*. Mussel growth across sites was positively associated with Chl *a*, particulate organic carbon (POC), and particulate organic nitrogen (PON) but negatively associated with stoichiometric mismatch. Overall, as the elemental ratios of consumers became more different from those of their resources, growth declined, likely due to the energetic cost associated with processing lower quality food. Furthermore, the effect of food quantity on growth depended on stoichiometric mismatch. In New Zealand, where mismatch was high—i.e., consumer C:N differed substantially from resource C:N—consumer growth was strongly affected by resource quantity (Chl *a* or POC). However, in Oregon, where mismatch was low, the relationship between resource quantity and growth was considerably weaker. This interaction between resource quantity and mismatch was not apparent for PON, which is consistent with variation in PON underlying variation in POM C:N and highlights the role of N in limiting growth. Previous research has neglected the importance of ecological stoichiometry as a mediator of consumer-resource interactions in rocky intertidal communities. I show that resource quality and quantity interact to determine consumer growth, highlighting the utility of ecological stoichiometry in understanding spatial subsidies in benthic marine systems.

## Introduction

Understanding consumer-resource interactions is a fundamental goal of community ecology. This is particularly true for interactions between autotrophs and herbivores, which can serve as a bottleneck limiting the movement of carbon to higher trophic levels (Cebrian, 1999). The emergence of the theory of ecological stoichiometry over the past 15–20 years has provided a new and insightful lens through which to view consumer-resource interactions (Elser et al., 2000; Sterner and Elser, 2002). Ecological stoichiometry highlights the importance of balancing the supply and ratios of a variety of substances (e.g., energy, nutrients) in ecological interactions. Thus, for example, it is not just carbon (C) as an energy currency that mediates the interaction between herbivores and autotrophs. Nutrient [e.g., nitrogen (N) or phosphorus (P)] limitation affects both consumer growth and the efficiency of foraging (Denno and Fagan, 2003; Hillebrand et al., 2009).

The C:N and C:P ratios of consumers are often different from those of the resources they consume. This is particularly true for herbivores and autotrophs; C:N and C:P ratios of herbivores are typically much lower than the ratios of the autotrophs on which they feed (Elser et al., 2000). The magnitude of this stoichiometric mismatch between herbivores and autotrophs can mediate herbivore growth and consumption rates (Urabe and Sterner, 1996; Hillebrand et al., 2009). Ecological stoichiometry therefore highlights an important aspect of consumer-resource interactions: both the quantity and the quality of available resources matter to consumers (Frost and Elser, 2002).

Ecological stoichiometry takes an ecosystem-level approach to understanding community-level processes, allowing investigators to infer mechanism based on the balancing of elemental supply and ratios. For example, this perspective has been invoked to characterize the consumption of diverse microbial resources [e.g., particulate organic matter (POM) in aquatic environments] by larger organisms in the absence of detailed taxonomic information on POM composition (Elser et al., 2000). Determining C:N, C:P, and N:P ratios of the available POM—which consists of a mixture of microorganisms and detritus—can provide insights into the potential for those resources to meet the nutritional needs of consumers (Sterner and Elser, 2002). Here, this perspective is used to clarify how variation in the quantity and quality of available food mediates the growth of suspension feeders on rocky shorelines.

I specifically studied how stoichiometric mismatch—differences in the C:N of consumers and resources—affected the growth rates of mussels (*Mytilus californianus* and *M. galloprovincialis*) on rocky shorelines. Mussels are important resources at the bases of intertidal food webs (Paine, 1974; Navarrete and Menge, 1996; Menge et al., 2003), thereby mediating subsidies from nearshore pelagic to intertidal benthic ecosystems. Mytilid mussels, which are common inhabitants of temperate intertidal habitats worldwide (Seed, 1969; Koehn, 1991), capture POM from the nearshore ocean (Ward et al., 1998), and the quantity and quality of the resources available to these consumers are mediated by coastal oceanographic processes operating at meso-scales (e.g., sites separated by up to 100 km within a region) and macro-scales (e.g., upwelling vs. downwelling regimes spanning 100 s of km; Menge et al., 2003, 2004).

The coastlines of Oregon, USA, and the South Island of New Zealand, where I conducted my measurements, are characterized by gradients in oceanographic processes that underlie variation in POM quality and quantity. The Oregon coast, part of the California Current System, experiences strong, but intermittent, upwelling of cold, nutrient-rich water during the summer months (Huyer, 1983); the strength of upwelling increases from north to south (Menge et al., 2004; Broitman et al., 2008). Variation in the quality and quantity of POM around the South Island of New Zealand is primarily associated with two contrasting oceanographic regimes: a weak intermittent upwelling region on the west coast and a persistent downwelling region on the east coast (Stanton, 1976; Menge et al., 2003). At more local scales, availability of POM and phytoplankton is influenced by the interactions between upwelling and meso-scale attributes such as terrestrial and riverine inputs (Hill and Wheeler, 2002; McLeod and Wing, 2009; Bracken et al., 2012), headlands (Jenks et al., 1982), and the width of the continental shelf (Menge et al., 1997).

Previous work in Oregon and New Zealand identified phytoplankton availability as an important determinant of mussel growth, explaining ≥49% of the variance in mussels' growth in C (Bracken et al., 2012). Phytoplankton are a high-quality food source, and mussels sort the bulk POM, which includes a substantial amount of low-quality, terrestrially-derived detritus (Bracken et al., 2012), preferentially retaining phytoplankton, and rejecting detritus (Ward et al., 1998). This selectivity is both imperfect and energetically costly, suggesting that both POM quality (i.e., stoichiometric mismatch) and quantity [i.e., particulate organic carbon (POC), particulate organic nitrogen (PON), or phytoplankton availability (Chl *a*)] could affect mussel growth. Note that these different “quantitative” aspects of the POM also represent differences in quality, with POC describing the availability of low-quality resources and PON and Chl *a* describing the availability of high-quality resources.

I measured mussel growth rates at sites along the coastlines of Oregon and New Zealand and evaluated growth as a function of resource availability and stoichiometric mismatch in C:N. In line with previous work evaluating effects of resource quality (e.g., C:P) and quantity (e.g., Chl *a*) on consumer biomass (Qin et al., 2007), I hypothesized that consumer growth would increase with increasing food quantity but decline with decreasing food quality. Thus, I predicted that mussel growth would be higher at sites characterized by higher Chl *a*, POC, and PON availability but lower at sites characterized by greater stoichiometric mismatch between mussels and POM.

## Materials and methods

### Study sites

**Figure 1:**
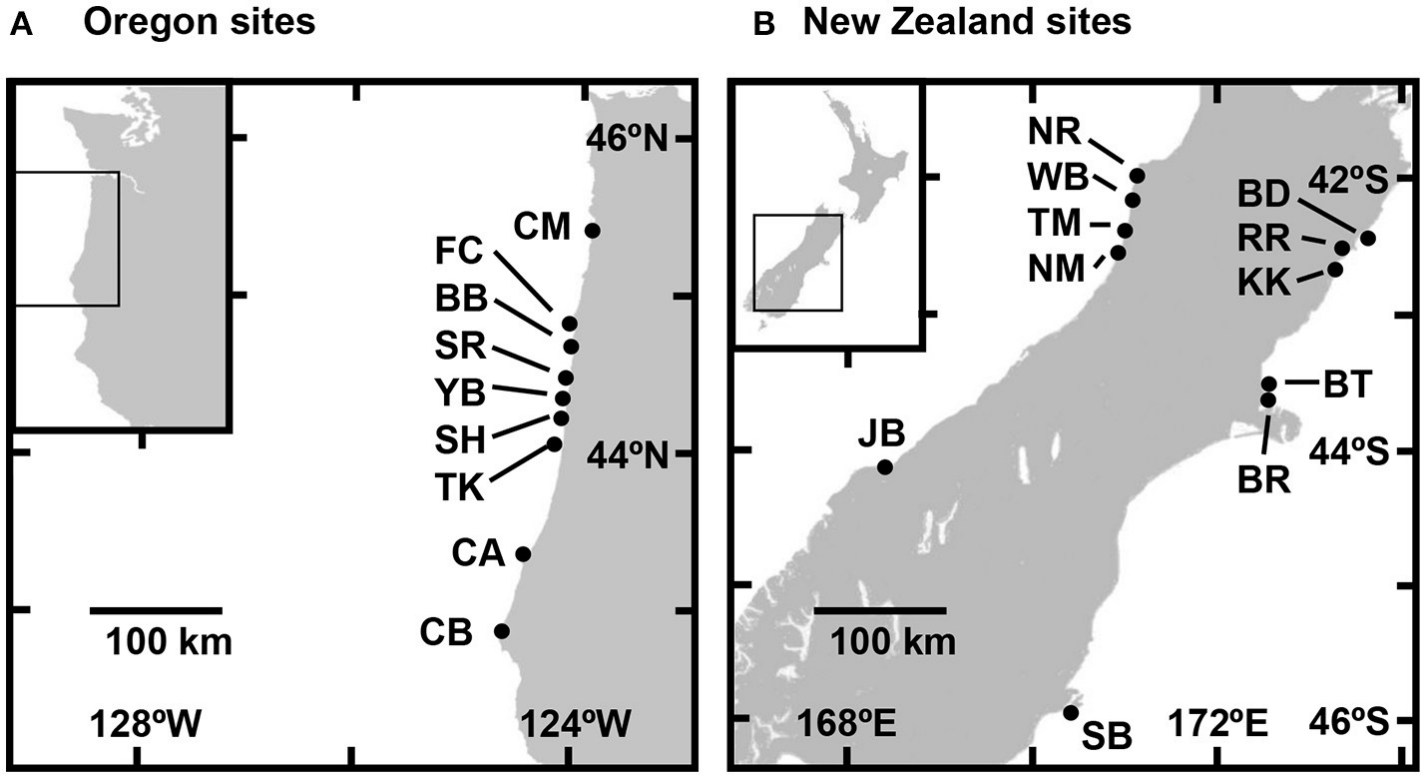
Sampling locations on the coasts of **(A)** Oregon, USA, and **(B)** South Island, New Zealand. See Table 1 for site abbreviations and characteristics.

Water-column attributes were quantified at nine sites along the coast of Oregon, USA, and eleven sites along the west and east coasts of the South Island of New Zealand (Table 1, Figure 1). Sites were all characterized by rocky reefs on exposed open coastlines that supported substantial populations of congeneric intertidal mussels (*M. californianus* in Oregon and *M. galloprovincialis* in New Zealand). Sites spanned 475 km of the Oregon coastline from Cape Meares (CM) in the north to Cape Blanco (CB) in the south (Figure 1A). The five sites on the west coast of the South Island spanned 325 km from the mouth of the Nile River (NR) in the north to Jackson Bay (JB) in the south, and the six sites on the east coast spanned 475 km from Blue Duck Creek (BD) in the north to Sandfly Bay (SB) on the Otago Peninsula in the south (Figure 1B).

**Table 1:** Characteristics of particulate organic matter (POM) and mussels at sites in Oregon, USA, and the South Island of New Zealand.

**Site**	**Chl *a* (μg L^−1^)**	**POC (μg L^−1^)**	**PON (μg L^−1^)**	**C:Chl *a* (μg μg^−1^)**	**POM C:N**	**Mussel C:N**	**Growth (mg g^−1^ yr^−1^)**
**(A) OREGON**							
Cape Meares (CM)	8.0	951.9	243.5	119.0	4.6	5.6	762.0
Fogarty Creek (FC)	6.2	860.5	181.2	139.3	5.5	5.3	960.3
Boiler Bay (BB)	5.4	757.6	179.0	140.3	4.9	5.3	757.0
Seal Rock (SR)	27.1	2150.9	422.0	79.3	5.9	5.7	1741.5
Yachats Beach (YB)	127.0	4565.3	1038.5	35.9	5.1	6.2	4082.6
Strawberry Hill (SH)	36.1	2569.0	598.2	71.1	5.0	5.7	1209.2
Tokatee Klootchman (TK)	43.8	2556.0	516.3	58.3	5.8	5.9	1927.6
Cape Arago (CA)	3.3	491.0	137.8	151.1	4.2	4.8	655.9
Cape Blanco (CB)	7.2	855.3	280.3	119.6	3.6	4.9	375.3
**(B) NEW ZEALAND**							
**West Coast**							
Nile River (NR)	2.1	1229.5	127.0	585.5	11.3	4.7	378.1
Woodpecker Bay (WB)	2.6	1072.2	118.5	412.4	10.6	4.6	706.3
Twelve Mile (TM)	1.9	1146.0	123.4	603.2	10.8	4.6	516.3
Nine Mile (NM)	3.4	1385.4	128.2	407.5	12.6	4.8	791.0
Jackson Bay (JB)	1.7	1370.0	136.4	805.9	11.7	4.7	301.7
**East Coast**							
Blue Duck (BD)	1.4	793.5	94.1	566.8	9.8	4.5	115.0
Raramai (RR)	0.7	512.6	56.4	732.3	10.6	4.5	88.9
Kie Kie (KK)	0.8	557.0	123.7	696.3	14.2	4.5	26.2
Box Thumb (BT)	1.8	787.9	93.5	437.7	9.8	5.1	389.4
Boulder Bay (BR)	1.9	757.4	92.0	398.6	9.6	5.2	210.2
Sandfly Bay (SB)	1.1	660.2	70.7	600.2	10.9	4.7	125.0

### Particulate organic matter

At each site, water samples were collected for measurement of POC (μg L^−1^), PON (μg L^−1^), and Chl *a* (μg L^−1^). Oregon sites were visited monthly during the boreal summer (May through September) of 2003, and New Zealand sites were visited twice per month during the austral spring and summer (October through January) of 2003 and 2004. Samples were collected by filling 3 acid-washed opaque high-density polyethylene brown plastic bottles at ~0.5 m depth in well-mixed water in the surf zone at each site at low tide. A 50 mL sample from each sample bottle was filtered through a 25 mm Whatman GF/F glass-fiber filter. These filters were transported to the laboratory on ice and analyzed for Chl *a* using a Turner Designs benchtop fluorometer after extraction in 90% HPLC acetone for 24 h at −20°C (Welschmeyer, 1994). Chl *a* was a reasonable proxy for phytoplankton concentrations based on relationships between Chl *a* and counts of phytoplankton and detritus in Lugol's-preserved water samples from a subset of sites. Analyses showed that Chl *a* concentrations were positively associated with phytoplankton cell counts [*R*^2^ = 0.68; *F*_(1, 14)_ = 10.9, *p* = 0.005] and were unrelated to the amount of detritus in water samples [*R*^2^ = 0.01; *F*_(1, 14)_ = 0.2, *p* = 0.675].

A 100 mL sample from each bottle was filtered through a pre-combusted 25 mm Whatman GF/F filter for analyses of POC and PON. These filters were transported to the laboratory on ice, dried (50°C for 72 h), acid fumed to remove carbonate, and analyzed for C and N using a PDZ Europa ANCA-GSL elemental analyzer at the UC Davis Stable Isotope Facility.

### Mussel growth and tissue analyses

Mussel growth rates were measured by marking five patches (20 × 20 cm) in the middle of the *Mytilus* zone at each site. The posterior edge of every mussel in every patch was carefully notched using a small triangular file. Mussels were collected ~1 year later, and both the initial length from the umbo to the notch and the final length from the umbo to the posterior edge were measured (Menge, 2000; Menge et al., 2008; Bracken et al., 2012). An average of 19 ± 2 (mean ± SE) mussels were recovered per patch. To determine the relationship between shell length and mass, additional un-notched mussels were collected from each site, spanning a wide range of shell lengths. The shell length (umbo to posterior edge) of each mussel was measured, and the tissue from each mussel was dried to constant mass at 50°C. These measurements were used to determine the relationship between dry tissue mass and shell length at each site, then that relationship was used to estimate the initial and final dry tissue mass of each marked mussel at each site. These conversions were necessary because length-mass relationships differed at the different sites. The average *in situ* biomass-specific growth rate of mussels at each site (mg dry tissue g^−1^ d^−1^) was calculated, then all values were normalized to an annual growth rate for each site (mg dry tissue g^−1^ yr^−1^) based on the exact number of days between notching and collection.

Whole mussel tissue (3 randomly-selected individuals from each site) was carefully dissected from the shells, dried to constant mass at 50°C, and ground to a fine powder. Samples were analyzed for C and N (%) using a PDZ Europa ANCA-GSL elemental analyzer at the UC Davis Stable Isotope Facility.

### Statistical analyses

Particulate and mussel C:N mass ratios were multiplied by 1.167 to calculate molar ratios. All values (water-column: Chl *a*, POC, PON, C:Chl *a*, POM C:N; mussels: C:N, annual growth rate) for each site were averaged, and each site was treated as a single data point for analyses, resulting in measurements from *N* = 20 sites (9 sites in Oregon and 11 sites in New Zealand; Table 1) spanning a range of oceanographic and hydrodynamic conditions (Bracken et al., 2012). This averaging was necessary because mussel growth could only be calculated as an annual average for each site.

Stoichiometric mismatch was calculated for each site as the proportional difference between the carbon-to-nitrogen ratios (C:N) of the mussels (C:N_*Consumer*_) and the POM (C:N_*Resource*_; Hillebrand et al., 2009):

$$\text{Mismatch}=\text{ln}\left(\frac{\text{C}:{\text{N}}_{Resource}}{\text{C}:{\text{N}}_{Consumer}}\right)$$

A positive mismatch value indicated that the resources at a site (i.e., POM) were characterized by higher C:N than the consumers (i.e., mussels), and a negative mismatch value indicated that the resources were characterized by lower C:N than the consumers. Log ratios are more effective at comparing C:N of resources and consumers than simple ratios because the natural log linearizes the ratio so that deviations in the numerator are equivalent to deviations in the denominator (Hedges et al., 1999).

General linear models (PROC GLM) and *t*-tests in SAS v. 9.4 (SAS Institute, Inc., Cary, North Carolina, USA) were used to evaluate relationships between site-level attributes after verifying assumptions of normality and homogeneity of variances. In particular, annual mussel growth rates were described as a function of resource quality (i.e., stoichiometric mismatch), resource quantity (i.e., Chl *a*, POC, or PON) a “quality × quantity” interaction (i.e., “Mismatch × Chl *a*,” “Mismatch × POC,” or “Mismatch × PON”), and region (i.e., Oregon vs. New Zealand; Tables 2A, 3A, 4A). Region was included as a factor in the models to account for geographic and taxonomic differences between Oregon and New Zealand. Additional analyses were conducted to evaluate relationships within each region separately, and these analyses evaluated growth as a function of stoichiometric mismatch and resource quantity (Chl *a*, POC, or PON; Tables 2B, 2C, 3B, 3C, 4B, 4C). We only included main effects in these models, as no “Mismatch × Quantity” interactions were statistically significant (*p* > 0.056 in all cases). Models used Type III sums of squares, so the effect of every factor was evaluated after accounting for all other factors in the model. In most cases where variances were not homogeneous, data were natural-log transformed (ln[*x*] or ln[*x*+1]). In some cases (e.g., comparisons between Oregon and New Zealand), heterogeneity of variances could not be corrected by transformation. These data were analyzed using generalized linear models (PROC GENMOD) in SAS v. 9.4, with log links and gamma distributions. All generalized linear models converged.

**Table 2:** Effects of phytoplankton availability and stoichiometric mismatch on annual growth rates of mussels.

**Source of variation**	**df**	**MS**	**Parameter estimates**	***F***	***p***
**(A) OVERALL**					
Stoichiometric mismatch	1	2.79	−2.70	15.34	0.001
Chl *a*	1	7.88	+0.84	43.3	<0.001
Mismatch × Chl *a*	1	5.29	+1.74	29.0	<0.001
Region (OR vs. NZ)	1	0.29	+0.73	1.6	0.230
Error	15	0.18			
**(B) OREGON**					
Stoichiometric mismatch	1	0.38	+1.78	5.0	0.066
Chl *a*	1	3.01	+0.54	39.7	<0.001
Error	8	0.08			
**(C) NEW ZEALAND**					
Stoichiometric mismatch	1	0.37	−1.39	1.8	0.213
Chl *a*	1	7.30	+0.98	35.9	<0.001
Error	10	0.20			

*Analyses are based on Type III Sums of Squares, so effects of each factor are determined after taking all other factors into account*.

**Table 3:** Effects of POC availability and stoichiometric mismatch on annual growth rates of mussels.

**Source of variation**	**df**	**MS**	**Parameter estimates**	***F***	***p***
**(A) OVERALL**					
Stoichiometric mismatch	1	2.80	−9.97	12.6	0.003
POC	1	5.00	+0.98	23.9	<0.001
Mismatch × POC	1	1.90	+1.15	10.7	0.005
Region (OR vs. NZ)	1	0.22	+1.14	<0.1	0.936
Error	15	0.24			
**(B) OREGON**					
					
Stoichiometric mismatch	1	0.28	+1.52	2.5	0.168
POC	1	2.80	+0.80	24.9	0.003
Error	8	0.11			
**(C) NEW ZEALAND**					
Stoichiometric mismatch	1	1.57	−2.78	6.8	0.030
POC	1	7.12	+2.38	31.7	<0.001
Error	10	0.22			

*Analyses are based on Type III Sums of Squares, so effects of each factor are determined after taking all other factors into account*.

**Table 4:** Effects of PON availability and stoichiometric mismatch on annual growth rates of mussels.

**Source of variation**	**df**	**MS**	**Parameter estimates**	***F***	***p***
**(A) OVERALL**					
Stoichiometric mismatch	1	1.75	−9.52	3.1	0.101
PON	1	3.89	+0.95	6.8	0.020
Mismatch × PON	1	1.40	+1.51	2.5	0.138
Region (OR vs. NZ)	1	0.72	+1.73	1.3	0.280
Error	15	0.24			
**(B) OREGON**					
Stoichiometric mismatch	1	0.74	+2.48	6.0	0.050
PON	1	2.73	+0.88	22.1	0.003
Error	15	0.25			
**(C) NEW ZEALAND**					
Stoichiometric mismatch	1	3.50	−4.45	5.3	0.050
PON	1	3.66	+2.31	5.6	0.046
Error	15	0.24			

*Analyses are based on Type III Sums of Squares, so effects of each factor are determined after taking all other factors into account*.

## Results

### Particulate organic matter and mussel characteristics

Despite spanning similar latitudes (Figure 1), water-column and mussel characteristics of sites on the coast of Oregon, USA, and the South Island of New Zealand differed substantially, creating a gradient in resource quantity and quality (Chl *a*, POC, PON, C:Chl *a*, POM C:N) and consumer condition (mussel C:N, mussel growth; Table 1). Overall, Chl *a* (GENMOD: Wald χ^2^ = 52.1, *p* < 0.001), POC (GENMOD: Wald χ^2^ = 7.2, *p* = 0.007), PON (GENMOD: Wald χ^2^ = 41.1, *p* < 0.001), mussel C:N (Wald χ^2^ = 27.7, *p* < 0.001), and mussel growth (Wald χ^2^ = 17.8, *p* < 0.001) were higher on the Oregon coast, whereas C:Chl *a* (Wald χ^2^ = 129.8, *p* < 0.001) and POM C:N (Wald χ^2^ = 381.5, *p* < 0.001) were higher on the coasts of the South Island (Table 1). PON availability was highly correlated with POC (*R*^2^ = 0.70) and Chl *a* (*R*^2^ = 0.94), and POC availability was highly correlated with Chl *a* (*R*^2^ = 0.73).

**Figure 2:**
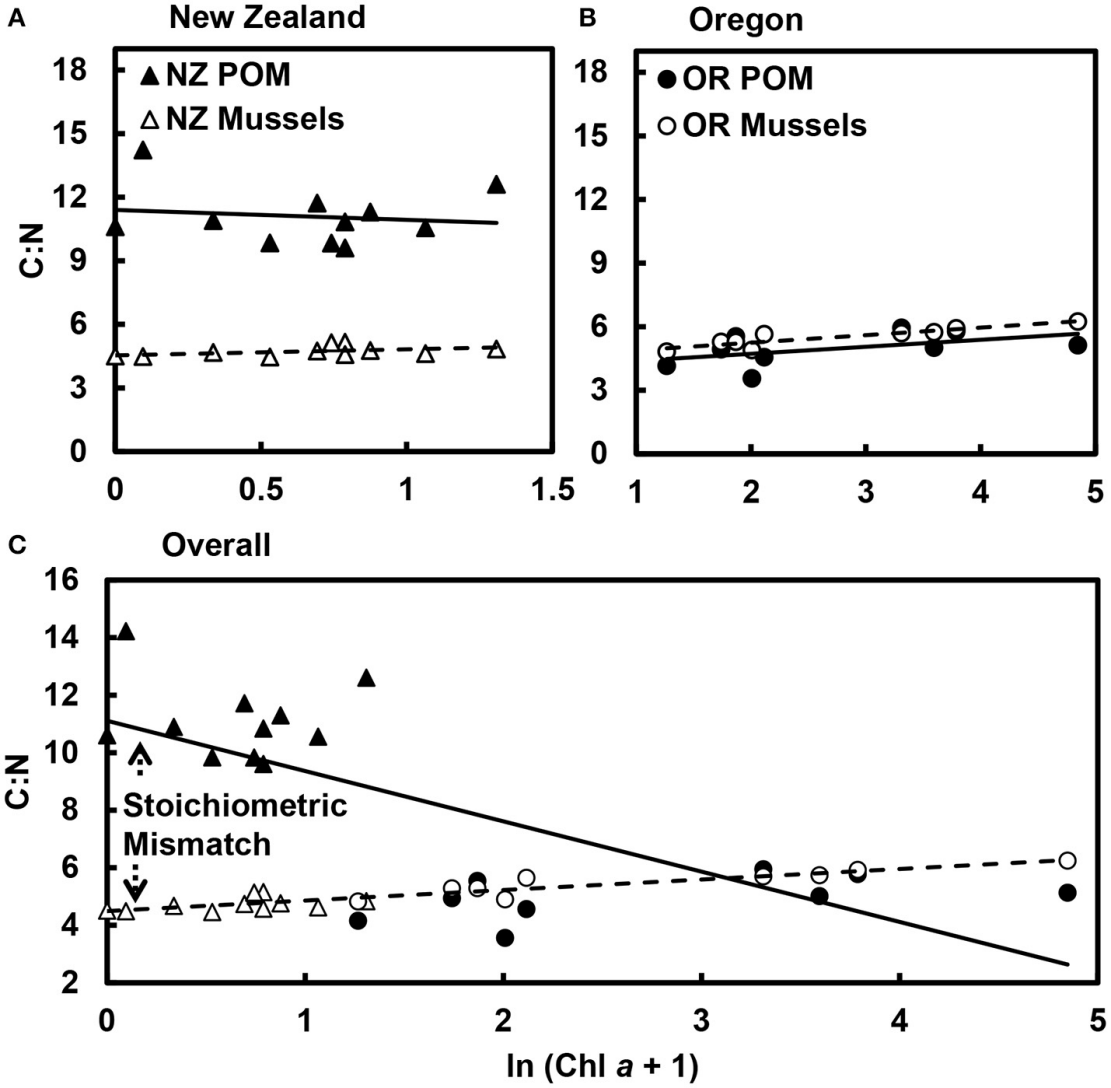
Stoichiometric mismatch in C:N between mussels and particulate organic matter (POM) declined as phytoplankton abundance increased. Mismatch was much more pronounced in **(A)** New Zealand (*p* < 0.001) than in **(B)** Oregon (*p* = 0.327). **(C)** Overall, as phytoplankton abundance (ln Chl *a*) increased, the C:N of the POM declined (*R*^2^ = 0.47, *p* < 0.001), whereas the C:N of the mussels increased (*R*^2^ = 0.85, *p* < 0.001), resulting in mismatch between consumers and their resources. Data points are mean values for each site.

Stoichiometric mismatch between mussels and POM was more pronounced in New Zealand (*t* = 5.8, df = 10, *p* < 0.001; Figure 2A) than in Oregon, where there was little to no difference in the C:N of mussels and POM (*t* = 1.1, df = 8, *p* = 0.327; Figure 2B). Overall, as phytoplankton availability (Chl *a*) in the nearshore ocean increased, the carbon-to-nitrogen ratio (C:N) of the POM declined [GLM: *R*^2^ = 0.47; *F*_(1, 18)_ = 15.8, *p* < 0.001; Figure 2C]. Variation in the POM C:N was largely associated with variation in PON [GLM: *R*^2^ = 0.44; *F*_(1, 18)_ = 14.2, *p* = 0.001]; there was no relationship between POM C:N and POC availability [GLM: *R*^2^ = 0.08; *F*_(1, 18)_ = 1.6, *p* = 0.229]. Note, also, that the decline in the POM C:N with increases in Chl *a* represented a difference between New Zealand and Oregon. There was no relationship between Chl *a* and POM C:N in either New Zealand alone [*F*_(1, 9)_ = 0.1, *p* = 0.761; Figure 2A] or Oregon alone [*F*_(1, 7)_ = 2.4, *p* = 0.163; Figure 2B]. Multiple regression suggested that changes in POM C:N were associated with simultaneous changes in PON and POC in Oregon [PON: *F*_(1, 5)_ = 440.2, *p* < 0.001; POC: *F*_(1, 5)_ = 251.0, *p* < 0.001; Chl *a*: *F*_(1, 5)_ = 0.3, *p* = 0.636] and PON in New Zealand [PON: *F*_(1, 7)_ = 5.3, *p* = 0.055; POC: *F*_(1, 7)_ = 0.3, *p* = 0.591; Chl *a*: *F*_(1, 7)_ = 0.6, *p* = 0.465].

As Chl *a* increased, the C:N of the mussels increased [GLM: *R*^2^ = 0.85; *F*_(1, 18)_ = 103.1, *p* < 0.001; Figure 2] Within regions, this pattern held in Oregon [*F*_(1, 7)_ = 31.9, *p* < 0.001], but not in New Zealand [*F*_(1, 9)_ = 2.3, *p* = 0.164]. The difference in the relationships between Chl *a* and the C:N of the POM and mussels resulted in a divergence in the C:N of consumers and resources as Chl *a* increased, highlighting a decline in stoichiometric mismatch as the availability of high-quality food increased. Overall, C:N of the POM [coefficient of variation (c.v.) = 0.44] was much more variable than C:N of the mussels (c.v. = 0.11).

### Overall effects of resource quality and quantity on mussel growth

**Figure 3:**
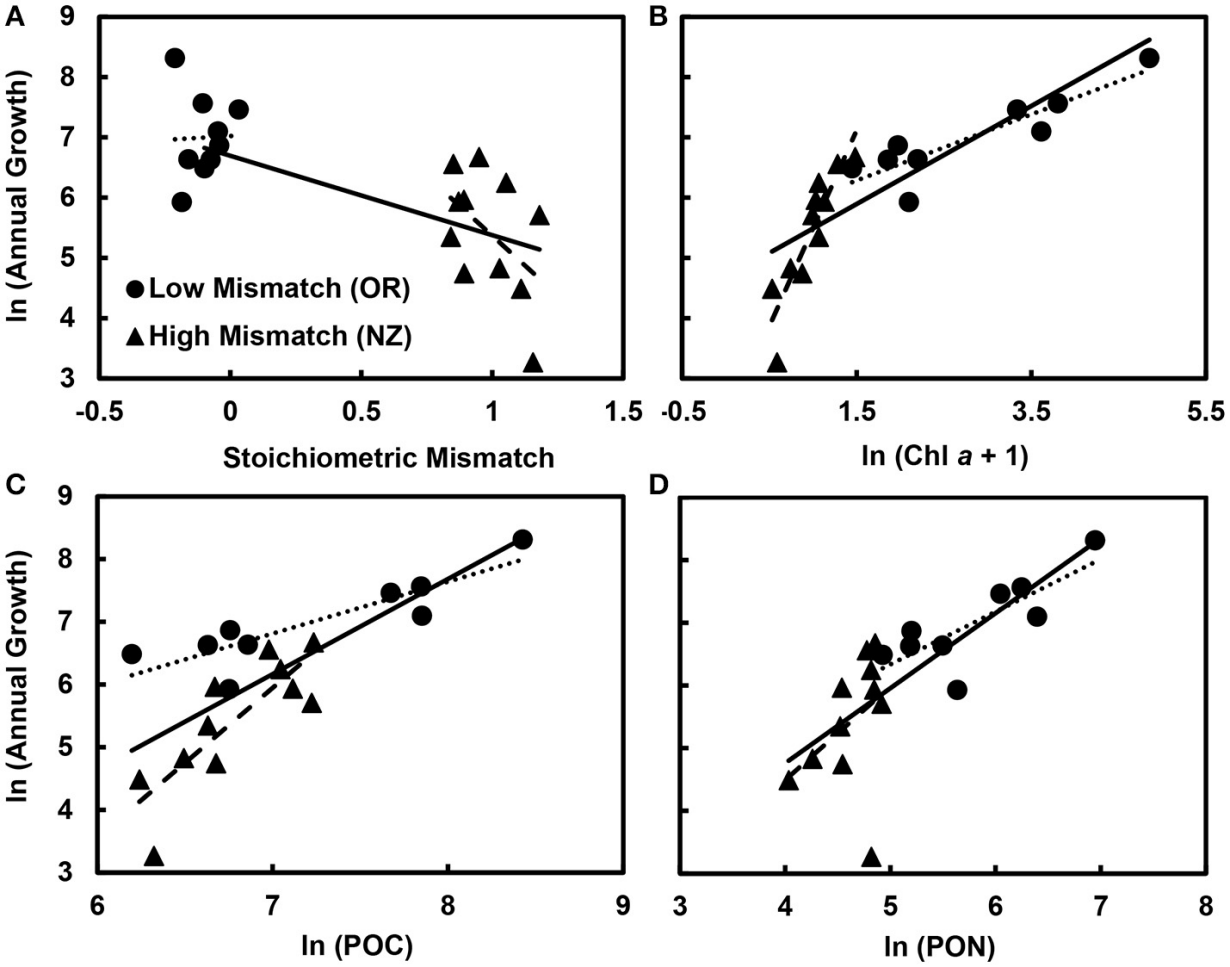
Effects of resource availability and stoichiometric mismatch on mussel growth. Across both regions, growth (originally measured in mg g^−1^ yr^−1^) declined with **(A)** stoichiometric mismatch (*p* = 0.001) and increased with **(B)** phytoplankton availability (Chl *a*, originally measured in μg L^−1^; *p* < 0.001), **(C)** particulate organic carbon (POC, originally measured in μg L^−1^; *p* < 0.001), and **(D)** particulate organic nitrogen (PON, originally measured in μg L^−1^; *p* < 0.001). Within regions, for **(B)** Chl *a* (“Mismatch × Chl *a*” interaction, *p* < 0.001) and **(C)** POC (“Mismatch × POC” interaction, *p* = 0.005), the effect of resource quantity on growth was stronger in New Zealand, where stoichiometric mismatch was high, than in Oregon, where mismatch was low, so the slopes differed. However, for **(D)** PON (“Mismatch × PON” interaction, *p* = 0.138), the effect of resource quality on growth did not differ between regions. Data points are mean values for each site. Solid trendlines indicate relationships across both regions, dotted trendlines indicate relationships across Oregon sites, and dashed trendlines indicate relationships in New Zealand.

Overall, stoichiometric mismatch negatively affected mussel growth rates (Figure 3A), but the strength of this relationship depended on the identity of the resource: Chl *a*, POC, or PON. After accounting for region (Oregon vs. New Zealand), Chl *a*, and the interaction between mismatch and Chl *a*, annual growth declined as mismatch increased [GLM: *F*_(1, 15)_ = 15.3, *p* = 0.001; Table 2A]. Similarly, after accounting for region, POC, and the interaction between mismatch and POC, growth declined as mismatch increased [GLM: *F*_(1, 15)_ = 12.6, *p* = 0.003; Table 3A]. However, after accounting for region, PON, and the interaction between mismatch and PON, there was little to no effect of mismatch on growth [GLM: *F*_(1, 15)_ = 3.1, *p* = 0.101; Table 4A].

Resource quantity enhanced growth, regardless of resource identity; mussel growth increased as Chl *a* [GLM: *F*_(1, 15)_ = 43.3, *p* < 0.001; Table 2A, Figure 3B], POC [GLM: *F*_(1, 15)_ = 23.9, *p* < 0.001; Table 3A, Figure 3C], and PON [GLM: *F*_(1, 15)_ = 6.8, *p* = 0.020; Table 4A, Figure 3D] increased. Furthermore, the effect of Chl *a* [GLM: “Mismatch × Chl *a*” interaction, *F*_(1, 15)_ = 29.0, *p* < 0.001; Table 2A, Figure 3B] and POC [GLM: “Mismatch × POC” interaction, *F*_(1, 15)_ = 10.7, *p* = 0.005; Table 3A, Figure 3C] on mussel growth depended on stoichiometric mismatch. The effects of both Chl *a* and POC availability on mussel growth were stronger (i.e., the slope of the relationship was steeper) in New Zealand, where mismatch was high, than in Oregon, where mismatch was low. In contrast, the effect of PON on growth was not affected by stoichiometric mismatch [GLM: “Mismatch × PON” interaction, *F*_(1, 15)_ = 2.5, *p* = 0.138; Table 4A, Figure 3D].

After accounting for the effects of stoichiometric mismatch, resource quantity, and the interaction between them, growth did not differ between Oregon and New Zealand. This lack of a difference between regions held regardless of resource identity: Chl *a* [*F*_(1, 15)_ = 1.6, *p* = 0.230; Table 2A], POC [*F*_(1, 15)_ < 0.1, *p* = 0.936; Table 3A], or PON [*F*_(1, 15)_ = 1.3, *p* = 0.280; Table 4A].

### Comparisons within oregon and New Zealand

Within each region, effects of resource quantity on mussel growth were universally positive. On the Oregon coast, growth increased with increases in Chl *a* [*F*_(1, 8)_ = 39.7, *p* < 0.001; Table 2B, Figure 3B], POC [*F*_(1, 8)_ = 24.9, *p* = 0.003; Table 3B, Figure 3C], and PON [*F*_(1, 8)_ = 22.1, *p* = 0.003; Table 4B, Figure 3D]. Similarly, in New Zealand, mussel growth was positively related to Chl *a* [*F*_(1, 10)_ = 35.9, *p* < 0.001; Table 2C, Figure 3B], POC [*F*_(1, 10)_ = 31.7, *p* < 0.001; Table 3C, Figure 3C], and PON [*F*_(1, 10)_ = 5.6, *p* = 0.046; Table 2C, Figure 3D].

Effects of stoichiometric mismatch on growth were neither as strong nor as consistent as effects of resource quantity, and they differed between regions. In Oregon, effects of mismatch on growth tended to be positive (Tables 2B, 3B, 4B, Figure 3A) and were generally weak [Chl *a*: *F*_(1, 8)_ = 5.0, *p* = 0.066; POC: *F*_(1, 8)_ = 2.5, *p* = =; PON: *F*_(1, 8)_ = 6.0, *p* = 0.050]. In contrast, effects of mismatch on growth of New Zealand mussels tended to be negative (Tables 2C, 3C, 4C, Figure 3A) and were more consistent than those in Oregon [Chl *a*: *F*_(1, 10)_ = 1.8, *p* = 0.213; POC: *F*_(1, 10)_ = 6.8, *p* = 0.030; PON: *F*_(1, 10)_ = 5.3, *p* = 0.050].

## Discussion

Mussels are common, often dominant, suspension feeders on temperate rocky shorelines (Seed, 1969; Paine, 1974; Koehn, 1991; Menge et al., 2003). They therefore play an essential role as mediators of subsidies from nearshore pelagic into intertidal benthic ecosystems (Bracken et al., 2012). Understanding the factors underlying these subsidies is therefore crucial to understanding energy and nutrient flows into and within rocky shore systems. Here, I show that it is not just the quantity (i.e., Chl *a*, POC, or PON; Figures 3B–D), but also the quality (i.e., stoichiometric mismatch; Figure 3A) of the available particulate organic matter that mediates mussel growth in open-coast intertidal systems. This is in line with my initial predictions and previous research (e.g., Qin et al., 2007; Rowland et al., 2015); I predicted that growth would be higher where Chl *a*, POC, and PON availability was greater and lower where stoichiometric mismatch was greater.

Note that mussel growth is also related to water temperature, declining when and where temperatures are cooler (Menge et al., 2008). I did not measure temperatures during this study, but New Zealand coastal waters are warmer than those on the Oregon coast, and there is substantial within-region temperature variability due to site differences in nearshore oceanographic conditions (Menge et al., 2003, 2004). However, the factors included in my statistical models explained most of the variance in mussel growth (*R*^2^ generally > 0.80), so temperature was not a strong mediator of growth relative to POM quality and quantity. Note also that many of the relationships between growth, mismatch, and POM quality and quantity were associated with differences between New Zealand sites (characterized by high mismatch) and Oregon sites (characterized by low mismatch). “Region” was included in statistical models to account for these differences, but it is important to note that differences between *M. californianus* and *M. galloprovincialis* could underlie some of the observed relationships. For example, *M. galloprovincialis* has been introduced to the coast of California, USA, where it now co-occurs with the native *M. californianus*. The introduced *M. galloprovincialis* grows more rapidly than its native congener under silty conditions, suggesting that it may be better adapted to poor-quality resources (Harger, 1970).

However, increases in stoichiometric mismatch were still associated with lower growth in New Zealand, particularly after accounting for the effects of POC and PON availability on growth (Tables 3C, 4C, Figure 3A). In contrast, relationships between mismatch and growth actually tended to be positive in Oregon. Thus, whereas effects of mismatch on growth were relatively weak within each region, parameter estimates were universally positive in Oregon but negative in New Zealand. There was a weak, positive effect of mismatch on mussel growth in Oregon, particularly after accounting for effects of PON availability. Stoichiometric mismatch at most sites on the Oregon coast was actually negative (Figures 2, 3A), meaning that mussels had higher C:N, on average, than the POM. An increase in mismatch therefore represented a shift toward balanced C:N of consumers and resources, which could also explain why mussel C:N increased with increasing Chl *a*. A similar result was found for mismatch in C:P of zebra mussels and POM in Swedish lakes; mussel tissue condition increased with increasing mismatch (Naddafi et al., 2009). Zebra mussels tended to have higher C:P than the POM, and increases in mismatch resulted in more balanced C:P of mussels and POM. The intriguing possibility that increases in mismatch could enhance growth if mismatch is negative would benefit from additional study, particularly in controlled mesocosm settings.

I did not, *a priori*, expect that the effect of resource quantity on consumer growth would depend on the stoichiometric mismatch between consumers and resources. If anything, I predicted that the effect of Chl *a* or POC on mussel growth would be greater where mismatch was lower. Previous work has shown that increases in food quantity have stronger effects on consumer growth where resource quality (e.g., C:P of algae) is high (Frost and Elser, 2002; Fink and Von Elert, 2006). In contrast, I found that increases in food quantity (Chl *a* or POC, but not PON) had stronger effects on mussel growth where resource quality was lower.

The interactions between resource quantity and quality described by Frost and Elser (2002) involved mayfly larvae feeding on benthic algae. In contrast, mussels are suspension feeders, feeding on POM in the water column, where assimilation efficiencies decline at high phytoplankton concentrations (Navarro and Winter, 1982). Scope for growth—the energy available for growth beyond that required for maintenance—in *Mytilus* is unimodally related to phytoplankton availability, declining at high concentrations due to optimization of assimilation efficiency at intermediate phytoplankton concentrations (Thompson and Bayne, 1974). The slope of the relationship between food availability and growth therefore tends to decline as Chl *a* concentrations increase (Bayne et al., 1989; Figure 3B). Because stoichiometric mismatch is closely related to Chl *a*—it declines with increasing phytoplankton availability (Table 1, Figure 2)—this translates to an interaction between mismatch and food availability (Figures 3B,C). Resource quantity at sites on the Oregon coast was exceptionally high, with average Chl *a* concentrations as high as 127 μg L^−1^ (Table 1), which likely overwhelmed the ability of mussels to feed effectively. The fact that the effect of PON on growth did not change with mismatch highlights two important aspects of these interactions: (1) growth is likely limited by N and (2) stoichiometric mismatch is associated with variation in PON, not POC.

These relationships between food quality, food quantity, and growth are common attributes of planktonic consumer-resource interactions in both marine and freshwater systems (Mitra and Flynn, 2007), so the patterns I describe here are not just limited to benthic suspension feeders. In general, the patterns documented here suggest that stoichiometric mismatch can affect the growth of intertidal suspension feeders, but they are based on observations, not experiments. A controlled mesocosm study, where POM C:N could be held constant and POM quantity varied, would clarify the roles of POM quality and quantity in mediating mussel growth.

It is clear from my work and that of others that ecological stoichiometry can provide important insights into the mechanisms underlying consumer-resource interactions (Frost and Elser, 2002; Hessen et al., 2002; Sterner and Elser, 2002; Fink and Von Elert, 2006; Mitra and Flynn, 2007). However, this perspective is seldom invoked to explain consumption of POM by benthic suspension feeders (but see Carmichael et al., 2004, 2012). Mytilid mussels, particularly edible species in the genus *Mytilus*, have long served as a model system in marine physiology, and a large body of work has explored how the quality and quantity of suspended particulate material affects the growth of mussels and other benthic suspension feeders (e.g., Bayne et al., 1989, 1993; Arifin and Bendell-Young, 1997; Carmichael et al., 2004, 2012; Bracken et al., 2012). However, most of that research has considered “quality” in terms of the amount of suspended organic vs. inorganic material. This is an important consideration, especially in coastal marine systems where suspension feeders must separate organic phytoplankton and detritus from inorganic silt and sand, but it has precluded a strictly stoichiometric perspective that examines seston quality in terms of elemental ratios in the organic fraction of the suspended particulate material.

I argue that our understanding of consumer-resource interactions in marine systems is incomplete without incorporating ecological stoichiometry. There is ample support for this perspective in phytoplankton-based open-water marine systems, where “stoichiometric modulation of predation” is an important component of models that explain the interactions between zooplankton and phytoplankton (Mitra et al., 2007). Models of climate-change impacts on pelagic marine systems explicitly invoke ecological stoichiometry to explain the effects of elevated CO_2_ and reduced nutrient availability on trophic transfer of energy (van de Waal et al., 2010). However, ecological stoichiometry is seldom used to explain the magnitudes of spatial subsidies into benthic marine systems, despite the roles that these systems have played as experimental models of consumer-resource interactions (e.g., Paine, 1992; Navarrete and Menge, 1996). Here, I show that stoichiometric mismatch mediates an important subsidy into intertidal ecosystems and determines the growth of a foundational basal species in those systems. The interaction between nearshore phytoplankton and onshore suspension feeders is not amenable to the manipulative field experiments that are a hallmark of rocky intertidal research. However, understanding this spatial subsidy is key to our understanding of the dynamics of coastal marine systems (Menge et al., 2003).

More generally, it is not just the quantity, but also the quality, of resources that is important in determining trophic transfer of energy and the growth of consumers. My research, and that of others, demonstrates that resource quantity and quality interact to influence consumption and assimilation across terrestrial, freshwater, and marine systems and highlights the utility of ecological stoichiometry in understanding these interactions (Frost and Elser, 2002; Denno and Fagan, 2003; Fink and Von Elert, 2006; Mitra et al., 2007; Hillebrand et al., 2009).

## Author contributions

MB conceived and designed the work; acquired, analyzed, and interpreted data; and drafted the manuscript. MB therefore agrees to be accountable for all aspects of the work in ensuring that questions related to the accuracy or integrity of any part of the work are appropriately investigated and resolved.

### Conflict of interest statement

The author declares that the research was conducted in the absence of any commercial or financial relationships that could be construed as a potential conflict of interest.
